# Surgical Outcome of Idiopathic Epiretinal Membranes with Intraretinal Cystic Spaces

**DOI:** 10.1371/journal.pone.0168555

**Published:** 2016-12-19

**Authors:** Yusuke Shiode, Yuki Morizane, Shinji Toshima, Shuhei Kimura, Fumiaki Kumase, Mio Hosokawa, Masayuki Hirano, Shinichiro Doi, Kosuke Takahashi, Mika Hosogi, Atsushi Fujiwara, Fumio Shiraga

**Affiliations:** Department of Ophthalmology, Okayama University Graduate School of Medicine, Dentistry and Pharmaceutical Sciences, Okayama City, Okayama, Japan; Massachusetts Eye & Ear Infirmary, Harvard Medical School, UNITED STATES

## Abstract

**Objective:**

To investigate the occurrence ratio, localization, and surgical outcomes of intraretinal cystic spaces in idiopathic epiretinal membranes (ERMs).

**Methods:**

We retrospectively reviewed the charts of 432 eyes of 398 consecutive patients with idiopathic ERM who underwent vitrectomy and ERM peeling from January 2012 to September 2015. We selected cases with intraretinal cystic space prior to surgery, detected by spectral-domain optical coherence tomography. We then evaluated the effects of ERM peeling on intraretinal cystic spaces, best corrected visual acuity, and central retinal thickness at 6 months after surgery.

**Results:**

Twenty-four eyes (5.5%) showed intraretinal cystic spaces before surgery, present in the inner retinal layer (the inner group) in 9 eyes, in the outer retinal layer (the outer group) in 6 eyes, and in both the inner and the outer retinal layers (the combined group) in 9 eyes. Additionally, 30 eyes with ERM but without any presence of intraretinal cystic space were selected randomly and classified as the no cyst group. At 6 months after surgery, the disappearance rate of cystic spaces was significantly greater for the outer group than for the inner group (83.3% and 11.1%, respectively, P = 0.011). The mean best corrected visual acuity improved significantly after surgery in the inner group, the outer group, and the no cyst group (P < 0.05 for all three groups) but did not improve in the combined group (P = 0.58). The mean central retinal thickness decreased significantly after surgery in the inner group, the combined group, and the no cyst group (*P* < 0.05).

**Conclusions:**

Intraretinal cystic spaces were observed in 5.5% of preoperative idiopathic ERM cases. Following surgery, the cystic spaces in the outer retinal layer disappeared at higher rates than those in the inner retinal layer, suggesting that the pathophysiologies of these cystic spaces are different.

## Introduction

Epiretinal membrane (ERM) is the most common type of fibrocellular proliferation found on the inner limiting membrane (ILM) and is significantly associated with aging [1–3]. Although patients with ERM can be completely asymptomatic when the membrane is thin, its progression to a thick and contractile membrane may result in macular distortion, thus inducing metamorphopsia and loss of central visual function. Pars plana vitrectomy and ERM peeling with or without ILM removal are the only treatments for ERM [4–6]. However, even when ERM peeling is performed, the rate of postoperative visual acuity improvement is only 50–80%, and metamorphopsia remains in a high percentage of patients [6–9]. Although there have been many studies on visual acuity prognosis following ERM surgery, it is unknown what factors other than preoperative visual acuity and duration of symptoms are related to visual prognosis [8, 10, 11].

The advent of optical coherence tomography (OCT) has made it possible to observe the intraretinal space in a variety of eye diseases [12–16], including diseases in the intraretinal cystic space. These diseases are divided into two main types. One is cystoid macular edema (CME), which is observed in cases of diabetic retinopathy [17], vascular occlusion [18], and following cataract surgery [19]. CME is thought to increase vascular permeability mainly due to ischemia and inflammation. It is observed both in the inner and outer layers of the retina and causes loss of central visual function. The second type is known as microcystic macular edema (MME). In OCT images, MME is observed in the inner nuclear layer (INL) as lacunar areas of hyporeflectivity with clear boundaries and an absence of a clearly visible cell wall. Originally, MME was considered to occur mainly in patients with optic neuritis and optic atrophy and to be due to retrograde synaptic degeneration leading to Müller cell dropout [15, 20]. Recently, however, it has been reported that MME is not a specific feature of optic neuropathy but rather a nonspecific OCT finding seen in various retinal diseases including age-related macular degeneration, central serous chorioretinopathy, and ERM [15].

It has been reported that cystic space can be observed both before and after ERM surgery [21–23]. Investigations of cases in which INL cystic spaces, indicating MME, occurred after ERM surgery have shown that it is possible that cystic spaces can be caused by ILM peeling and cataract surgery [21–23]. However, the incidence and clinical significance of cystic spaces observed prior to ERM surgery remain unknown. In this study, we investigated the hypothesis that preoperative cystic spaces are related to ERM postoperative visual acuity prognosis by conducting a retrospective investigation of the preoperative spectral domain OCT of 432 eyes of 398 patients with ERM who underwent primary pars plana vitrectomy with membrane peeling, and we examined the incidence of cystic space in the various retinal layers, as well as the effects of membrane peeling on MME, visual acuity, and retinal thickness.

## Patients and Methods

### Study design and patients

We retrospectively reviewed the charts of a consecutive series of 432 eyes of 398 patients with idiopathic ERM who underwent primary pars plana vitrectomy with membrane peeling. All patients were treated in Okayama University Hospital between January 2012 and September 2015. This study was approved by the Institutional Review Board of the Okayama University Hospital and in accordance with the tenets of the Declaration of Helsinki. Each patient was informed about the risks and benefits of the surgery and their written, informed consent was obtained.

In this study, all cases were idiopathic ERMs. ERMs secondary to other ocular pathologies (e.g., diabetic retinopathy, vascular occlusion, ocular trauma, retinal detachment, high myopia and uveitis) were excluded. Furthermore, patients with systemic disorders (e.g., uncontrolled diabetes or hypertension) and / or past history of life-threatening diseases (e.g., cardiac infarction or cerebrovascular disturbance) were excluded from the study.

### Ophthalmic examinations

All patients underwent comprehensive ophthalmologic examinations before and 6 months after surgery. These examinations included measurement of best-corrected visual acuity (BCVA) with refraction using the 5-m Landolt C acuity chart as well as indirect and contact lens slit-lamp biomicroscopy. All eyes were examined by spectral-domain optical coherence tomography using a commercially available instrument (Cirrus; Carl Zeiss Meditec, Inc., Dublin, California) before and 6 months after surgery. Five raster line scans (6 mm in length) centered on the fovea were obtained.

### Main outcome measures

The main outcome measures were the effects of ERM peeling on the intraretinal cystic spaces, BCVA, and central retinal thickness (CRT) before and 6 months after surgery.

### Surgical procedure

All patients underwent 25-gauge pars plana micro incision vitrectomy. Triamcinolone acetonide was used intraoperatively in all eyes in order to facilitate visualization of the vitreous and posterior hyaloid. The ERM as well as the ILM were peeled in the macular area using end-gripping forceps. The ILM was stained with 0.25 mg/ mL brilliant blue G solution (Coomassie BBG 250; Sigma-Aldrich, St. Louis, Missouri, USA) or 0.05 mg/mL indocyanine green solution (Diagnogreen Injection, Daiichi Pharmaceutical, Tokyo, Japan). Cataract extraction with posterior chamber intraocular lens implantation was performed before the pars plana vitrectomy in all cases with cataract.

### Statistical analysis

BCVAs were recorded as decimal values and converted to the logarithm of the minimal angle of resolution (logMAR) for statistical analysis. All visual acuity results are presented as the logMAR units with Snellen equivalent in parentheses. All statistical analyses were performed using SPSS ver. 22.0 for Windows (IBM, Armonk, NY). P values less than 0.05 were considered significant. Data are presented as mean ± SD. All statistical methods are specified in the relevant sections of the Results.

## Results

### Classification of the intraretinal cystic spaces and preoperative demographic data

**Fig 1:**
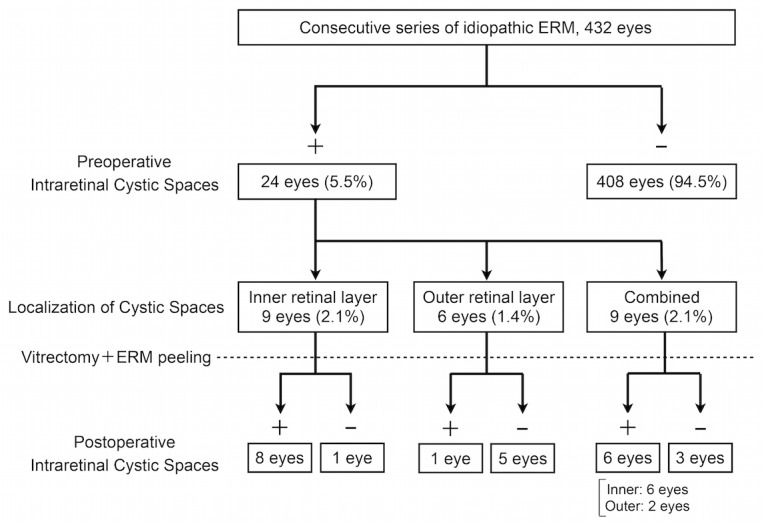
Flow Chart of Idiopathic ERM Case Selection and Follow-up in 432 Eyes. *ERM*, epiretinal membrane.

**Fig 2:**
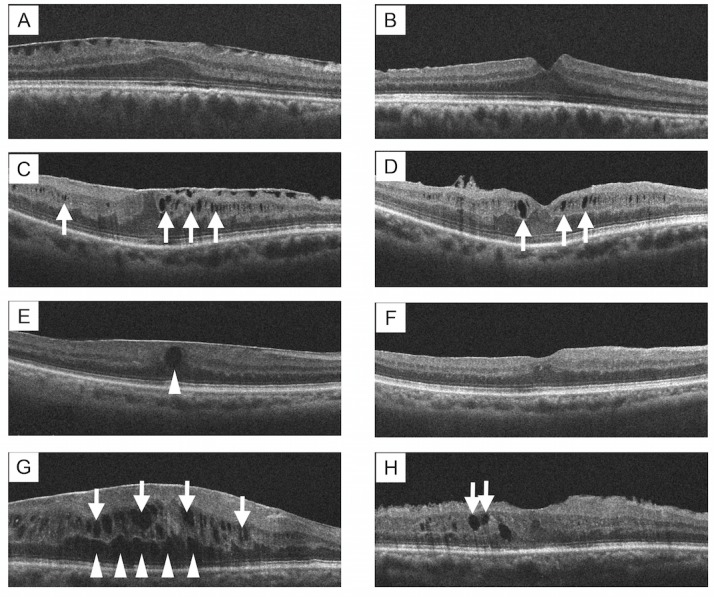
Representative ERM Cases of Each Group by Intraretinal Cyst Location. Panels (A–B) show examples of images from the group with no cysts (i.e., the no cyst group): A; pre-operation, B; post-operation. Panels (C–D) show examples of cases from the group with cystic spaces in the inner retina (i.e., the inner group): C; pre-operation, D; post-operation. Cystic spaces (*arrows*) partially remained after surgery. Panels (E–F) show examples of cases from the group with cystic spaces in the outer retina (i.e., the outer group): E; pre-operation, F; post-operation. Cystic spaces in the Henle layer (*arrowhead*) disappeared completely after surgery. Panels (G–H) show examples of cases from the group with cystic spaces in both the inner and outer retina (i.e., the combined group): G; pre-operation, H; post-operation. Some cystic spaces in the inner retinal layers (*arrows*) remained after surgery. *ERM*, epiretinal membrane.

Intraretinal cystic spaces were identified in 24 out of 432 eyes (5.5%). These 24 eyes were classified into three groups according to the localization of the cystic spaces (Fig 1). The inner group included 9 eyes with intraretinal cystic spaces present in the inner retinal layer, mainly in the inner nuclear layer. The outer group included 6 eyes with intraretinal cystic spaces present in the outer retinal layer, mainly in Henle’s layer or the outer plexiform layer. Finally, the combined group included 9 eyes with intraretinal cystic spaces present in both the inner and the outer retinal layers. As a control group, 30 eyes were randomly selected from the 408 eyes with no intraretinal cystic spaces and were classified as the no cyst group. Representative cases from each group are shown in Fig 2. Preoperative demographic data of all groups are shown in Table 1. Prior cataract surgeries were performed in 3 eyes (33.3%) of the inner group, 1 eye (11.1%) of the combined group, and 2 eyes (6.7%) of no cyst group. These cataract surgeries were performed at least one month before the vitreous surgery (range from one month to ten years) and none of the patients had any complications during cataract surgeries. In the all other cases, cataract surgery and vitreous surgery were performed simultaneously. We used BBG to stain the ILM of all cases except 2 eyes (22.2%) of the inner group, 2 eyes (22.2%) of the combined group and 12 eyes (40.0%) of no cyst group. In these 14 eyes, ICG were used to stain the ILM.

**Table 1:** Baseline Demographics and Clinical Data for All Groups.

	No cyst group	Inner group	Outer group	Combined group
	(30 eyes)	(9 eyes)	(6 eyes)	(9 eyes)
Age (years)	68.8 ± 6.4	72.4 ± 4.4	71.7 ± 5.5	67.4 ± 6.7
Sex (male / female)	12 / 18	2 / 7	5 / 1	6 / 3
LogMAR BCVA	0.179 ± 0.15	0.285 ± 0.26	0.223 ± 0.15	0.456 ± 0.33*
(Snellen equivalent)	(20/32)	(20/39)	(20/32)	(20/52)
CRT (μm)	410 ± 83.5	501 ± 56.4	391 ± 46.2	554 ± 134.3*
Cases with disruption of EZ (%)	3.3	11.1	0	33. 3*
Cases with disruption of ELM (%)	3.3	0	0	33. 3*

*LogMAR*, logarithm of the minimum angle of resolution; *BCVA*, best-corrected visual acuity; *CRT*, central retinal thickness. *EZ*, Ellipsoid zone. *ELM*, External limiting membrane. All data are presented as means ± SD.

Asterisk represents a significant difference (P < 0.05) from the no cyst group.

**Fig 3:**
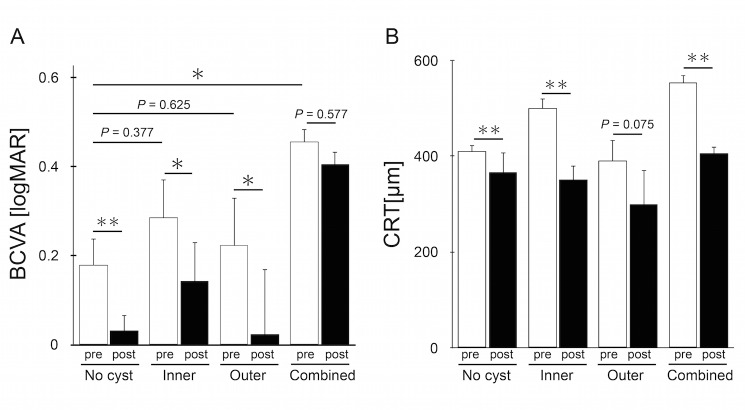
Comparison of Visual Acuity and Central Retinal Thickness at Baseline and at 6 months After Vitrectomy. A: Best-corrected visual acuities (BCVA) in logMAR value in all groups at baseline and 6 months after surgery. *logMAR*, logarithm of the minimum angle of resolution. B: Central retinal thickness (CRT) in all groups at baseline and 6 months after surgery. *P < 0.05, **P < 0.01.

The mean preoperative visual acuity was significantly lower in the combined group than in the no cyst group (P = 0.011, Mann-Whitney U test). The mean preoperative visual acuity in the inner group and the outer group showed no significant differences compared with the no cyst group (Fig 3A). In the combined group, 33.3% of cases showed ellipsoid zone (EZ) disruption, and 33.3% of cases showed external limiting membrane (ELM) disruption. This was significantly higher than the 3.3% of cases showing EZ disruption and the 3.3% of cases showing ELM disruption seen in the no cyst group (P = 0.032 and P = 0.032, respectively, Fisher's exact test).

### Postoperative changes in the intraretinal cystic spaces

At 6 months after surgery, intraretinal cystic spaces had completely disappeared in 1 out of 9 eyes (11.1%) in the inner group, 5 out of 6 eyes (83.3%) in the outer group, and 3 out of 9 eyes (33.3%) in the combined group (Fig 1). The disappearance rate of cystic spaces for the outer group was significantly higher than the disappearance rate for the inner group (P = 0.011, Fisher's exact test). Of the 6 eyes in the combined group with cystic spaces remaining after surgery, 3 eyes showed cystic spaces remaining in the outer retinal layer, while all 6 eyes showed cystic spaces remaining in the inner retinal layer.

### Postoperative changes in BCVAs

At 6 months after surgery, the mean logMAR BCVA (Snellen equivalent) was 0.032 (20/21) in the no cyst group, 0.143 (20/28) in the inner group, 0.023 (20/21) in the outer group, and 0.406 (20/51) in the combined group (Fig 3A). The mean BCVA improved significantly after surgeries in the no cyst group (P < 0.001, paired t-test), inner group (P = 0.021, paired *t*-test), and outer group (P = 0.028, paired *t*-test), while the combined group did not show significant improvement (P = 0.577, paired t-test).

### Postoperative changes in CRTs

At 6 months after surgery, the mean CRT was 366.3 μm for the no cyst group, 350.4 μm for the inner group, 299.3 μm for the outer group, and 406.6 μm for the combined group (Fig 3B). There were significant reductions in CRT after surgery for the no cyst group (*P* = 0.001, paired *t*-test), the inner group (*P* = 0.007, paired *t*-test), and the combined group (*P* = 0.005, paired *t*-test).

## Discussion

To our knowledge, the present study is the first to report the frequency of occurrence of intraretinal cystic spaces by retinal layer in naive ERM patients and to investigate the effects of ERM peeling on intraretinal cystic spaces. In this study, the occurrence sites of the intraretinal cystic spaces were divided into inner retina, outer retina, and combined inner and outer retina. We report that ERM peeling leads to cystic spaces in the outer retina disappearing in a high percentage of cases (83.3%), while cystic spaces in the inner retina disappear in only 11.1% of cases. We believe this difference is caused by differences in the pathology of cystic spaces in the outer retina and cystic spaces in the inner retina.

As shown in Fig 2E and 2G, cystic spaces in the outer retina were observed in Henle’s fiber layer and the outer plexiform layer. Both of these layers show a loose arrangement of nerve fibers, which allows an accumulation of fluid leaking from perifoveal capillaries [24]. Although we were unable to confirm the presence of leakage using fluorescein angiography in this study, the fact that the cystic spaces disappeared after surgery suggests that cystic spaces in the outer layer may be caused by perifoveal capillary leakage due to the increased mechanical traction caused by ERM [25]. In contrast, the cystic spaces of the inner retina were observed in the INL of the perifoveal area and showed complete disappearance following ERM in a significantly lower percentage of eyes (Fig 2C). Since the INL is the location in which the Müller cell nuclei are present, it has been reported that cystic spaces in the INL seen in various eye diseases, including ERM, represents Müller cell death [15, 20, 21]. In ERM, Müller cell death can occur as a result of increased traction, suggesting that the cystic spaces in the INL are less likely to disappear even if the traction is removed via ERM peeling. In this study, the 9 eyes in which cystic spaces were present in both the inner and outer retina were classified as “combined.” The cystic spaces remained in 6 of the 9 cases in spite of ERM peeling. The cystic spaces remained in the inner retina in all 6 cases with cystic spaces remaining post-surgery, and cystic spaces remained in the outer retina in just 2 eyes. This result is consistent with results that have shown that cystic spaces in the inner retina are likely to remain.

In this study, we report an incidence rate for intraretinal cystic spaces in untreated idiopathic ERM of 5.5%. This is lower than previously reported incidence rates, which range from 13.6% to 24.2% [6, 22, 23, 26]. One reason for this divergence may be differences in both ERM severity and duration of time since ERM onset. As discussed above, cystic spaces in both the inner and outer retina are thought to be produced by an increase in retinal traction caused by ERM. Thus, intraretinal cystic spaces are more likely to form both in cases with stronger traction from the ERM as well as in cases with a longer duration since ERM onset. Differences in OCT devices may also account for the divergent incidence rates. Except for one study by Frisina et al. [22], previous studies have used time domain OCT to detect intraretinal cystic spaces. The scan rate (400 scans/sec) of time domain OCT is markedly slower than the scan rate (50,000 scans/sec) of spectral domain OCT, and the resolution is also markedly poorer [27, 28]. Thus, lower resolution time domain OCT is more likely to lead to the misidentification of various retinal changes as intraretinal cystic spaces.

In this study, we determined the presence of intraretinal cystic spaces using sectional views of the retina that pass through the fovea. However, this observation method makes it impossible to detect extrafoveal intraretinal cystic spaces, and therefore the incidence rate of intraretinal cystic spaces in this study may have been underestimated. The scan rate of OCT has increased in recent years, allowing reconstruction of en face images from 3D retinal images [29]. These en-face images can reveal the presence of intraretinal cystic spaces throughout the entire macular area; therefore, we believe that future studies using en-face images could make it possible to obtain more accurate incidence rates.

The results of this study indicate that in the combined group, in which there were cystic spaces in both the inner and outer retina, preoperative visual acuity was poorer in comparison to the other groups. Furthermore, postoperative visual acuity for the combined group alone did not significantly improve following surgery, in spite of the fact that did CRT declined significantly. In addition, in the combined group the percentage of discontinuity of EZ and ELM was 33.3%, which was significantly higher in comparison to the other groups. These results may be due to a higher mechanical traction on the retina by the ERM in the combined group, which would lead to irreversible structural damage to the outer retina. This is supported by the fact that the occurrence of preoperative discontinuity in the outer retina (i.e., EZ and ELM) was higher in the group with cystic spaces in the inner retina (the inner group) than in the group with cystic spaces in the outer retina (the outer group; 11.1% and 0.0% respectively). This result strongly suggests that there was stronger traction due to ERM in the inner group than in the outer group. Although further study with a larger number of patients is required, we showed that the degree of traction due to ERM and the visual acuity prognosis could be estimated based on the location of preoperative cystic spaces.

This study was limited by the fact that it was a retrospective study with a small sample size in each group. Furthermore, we did not investigate the contribution of vascular leakage to cystic spaces using fluorescein angiography. As our investigation of visual function was limited to visual acuity, we did not investigate metamorphopsia. However, in this study we determined the incidence rate of intraretinal cystic spaces in cases of untreated idiopathic ERM by site within the retina, and we elucidated the effect of ERM peeling on intraretinal cystic spaces using spectral domain OCT. In cases of intraretinal cystic spaces in both the inner and outer retina in particular, the therapeutic prognosis of ERM peeling may be poor. A prospective study with a larger number of patients and multimodal morphological evaluations needs to be conducted to confirm our results.
